# A lightweight deep learning framework for reliable microscopy-based diagnosis of cutaneous leishmaniasis

**DOI:** 10.1371/journal.pone.0344561

**Published:** 2026-03-17

**Authors:** Nisreen Osman E. Ahmed, Samuel Mwalili, Murtada K. Elbashir

**Affiliations:** 1 Department of Mathematics, Institute for Science, Technology and Innovation, Pan African University, Nairobi, Kenya; 2 Department of Statistics and Computer Science, Faculty of Mathematical and Computer Sciences, University of Gezira, Wad Madani, Sudan; 3 Department of Statistics and Actuarial Sciences, Jomo Kenyatta University of Agriculture and Technology, Nairobi, Kenya; 4 College of Computer and Information Sciences, Jouf University, Sakaka, Saudi Arabia,; 5 Faculty of Mathematical and Computer Sciences, University of Gezira, Wad Madani, Sudan; Tehran University of Medical Sciences, IRAN, ISLAMIC REPUBLIC OF

## Abstract

Cutaneous leishmaniasis (CL) is a neglected tropical and zoonotic disease affecting both human and animal health, for which microscopic examination of Giemsa-stained slides remains the diagnostic reference standard despite being time-consuming and operator-dependent. In this study, we developed a lightweight, calibration-aware deep learning framework for automated amastigote detection and slide-level diagnostic probability estimation from microscopy images. A U-Net architecture with a MobileNetV2 encoder was employed for pixel-level parasite segmentation using a weakly supervised pseudo-labeling strategy on a single-center dataset comprising 292 field-of-view images. Slide-level diagnostic probabilities were derived via probability pooling and subsequently refined using post-hoc calibration techniques, including isotonic regression, Platt scaling, and temperature scaling. Model performance was evaluated on an independent test set using segmentation metrics (Dice coefficient and Intersection-over-Union) and diagnostic reliability metrics (AUROC, Brier score, and Expected Calibration Error). The proposed framework achieved a Dice coefficient of 0.901 and an IoU of 0.820 for segmentation against pseudo-label references, with strong discriminative performance at the slide level (AUROC = 0.978). Isotonic regression markedly improved probability reliability, reducing the Brier score from 0.089 to 0.030 and the Expected Calibration Error from 0.120 to 0.023 without significantly affecting discrimination. Statistical analyses confirmed the robustness of the calibration improvements. Overall, the results demonstrate that isotonic calibration enhances the interpretability and reliability of deep-learning-based CL diagnostics. The proposed lightweight framework provides a robust foundation for microscopy-based screening and supports future validation across broader datasets and One Health–oriented diagnostic applications.

## Introduction

Cutaneous leishmaniasis (CL) is a vector-borne zoonotic disease caused by protozoan parasites of the genus *Leishmania* and transmitted to humans and animals through the bite of infected phlebotomine sandflies, of which more than 90 vector species have been described [1,2]. More than 20 *Leishmania* species infect humans and other mammals, resulting in three principal clinical forms: cutaneous (CL), mucocutaneous (MCL), and visceral leishmaniasis (VL) [2,3]. Globally, an estimated 600,000–1,000,000 new CL cases occur annually; however, only a fraction are officially reported, reflecting substantial underdiagnosis and underreporting in endemic regions [2,4].

Cutaneous leishmaniasis is the most prevalent form of the disease in both humans and animal reservoirs. Lesions typically develop at the sandfly bite site, beginning as papules that enlarge, crust, and frequently ulcerate, often resulting in permanent scarring with significant functional, psychological, and social consequences [5,6]. Approximately 95% of CL cases occur in the Americas, the Mediterranean basin, the Middle East, and Central Asia [2,7]. In endemic regions, domestic dogs and wild rodents act as major reservoirs, sustaining transmission cycles and reinforcing the zoonotic nature of CL within the One Health framework. Accordingly, there is a pressing need for rapid, accurate, and scalable diagnostic tools that can support both human and animal health programs, particularly in resource-limited and field settings [8].

Microscopic examination of stained smears remains the reference standard for CL diagnosis in clinical laboratories, but it is labor-intensive, time-consuming, and highly dependent on operator expertise. Recent advances in computer vision and deep learning have motivated the automation of microscopy-based diagnostic workflows to reduce subjectivity and improve reproducibility. Although automated microscopy for CL has been less extensively studied than for malaria or Chagas disease, the underlying laboratory workflows are comparable, enabling adaptation of existing image-analysis pipelines. Traditional approaches typically rely on standardized image acquisition, color-space preprocessing (e.g., RGB or HSV), noise filtering, threshold-based segmentation, and rule-based classification, with deep neural networks increasingly incorporated when annotated datasets are available [9].

Early computational studies for CL microscopy adopted hybrid or multi-stage pipelines. For example, a U-Net-based segmentation approach followed by multi-class classification demonstrated the feasibility of automated parasite recognition in smear images [10]. Classical machine-learning methods have also been explored; a Viola–Jones detector with Haar-like features and AdaBoost achieved parasite-level sensitivity of 71% and specificity of 52% [11]. Beyond CL, lightweight convolutional encoders such as MobileNetV2 have demonstrated strong performance in dermatological image analysis, including high-accuracy skin cancer classification from dermoscopic images [12], highlighting their suitability for deployment in low-resource environments. Similarly, mobile-based deep-learning applications have achieved promising performance for presumptive CL diagnosis [13]. In addition, deep-learning analysis of clinical lesion photographs has shown strong diagnostic potential, with AlexNet differentiating CL from 26 other dermatoses with a mean accuracy of 95.0% on 2,458 images [14].

More recent studies have evaluated both deep-learning and classical approaches for CL microscopy. Comparative analyses of pretrained convolutional networks on ultrathin smears reported DenseNet201 outperforming EfficientNetB0, ResNet101, MobileNetV2, and Xception under cross-validation [15]. Other works have focused on parasite quantification and explainable neural networks to support antileishmanial drug discovery [16]. Additional segmentation and classification pipelines have reported variable performance, with sensitivities ranging from 72–92% and specificities from 65–99%, reflecting differences in datasets, staining protocols, and evaluation strategies [17–19]. Recent object-detection approaches using YOLO architectures trained on smartphone-acquired microscopy images have also shown promise across regional datasets [20,21].

While segmentation accuracy and diagnostic discrimination are widely reported, relatively few studies have systematically examined the *calibration* of deep-learning outputs, i.e., the agreement between predicted probabilities and observed outcome frequencies. Calibration is particularly important in diagnostic systems, where overconfident predictions may mislead clinical decision-making even when accuracy is high [22]. Recent work has demonstrated that post-hoc calibration methods such as isotonic regression, Platt scaling, and temperature scaling can substantially improve probability reliability in medical image segmentation without retraining [23–26]. These findings highlight the importance of explicitly evaluating and reporting calibration alongside conventional performance metrics to ensure trustworthy and interpretable diagnostic outputs.

Building on this literature, the present study introduces a lightweight, calibration-aware diagnostic framework that integrates a U-Net [27] architecture with a MobileNetV2 encoder [28]. This design prioritizes computational efficiency, reducing parameter counts and enabling potential deployment on mobile devices or in clinics with limited computing resources, without substantially compromising diagnostic accuracy, a critical requirement for resource-limited endemic regions. To mitigate the scarcity of expert pixel-level annotations, training masks were generated using an HSV-based color heuristic as a weakly supervised labeling strategy. It is important to note that this approach implies that the reported segmentation metrics primarily reflect agreement with these heuristic masks rather than a biologically verified ground truth, a limitation that is explicitly addressed in the discussion. Both pixel-level segmentation and slide-level diagnostic performance were evaluated, with particular emphasis on probability calibration using the Brier score, Expected Calibration Error, and reliability diagrams. The clinical implications of calibration, especially the trade-off between sensitivity and specificity, were further analyzed through operating-point sensitivity analyses and confidence intervals.

Although the present study is based on human microscopy images, cutaneous leishmaniasis is fundamentally a zoonotic disease involving interconnected human, animal, and environmental reservoirs. By improving the reliability and interpretability of microscopy-based diagnostics for human cases, the proposed framework contributes conceptually to One Health–oriented surveillance and control strategies. We explicitly acknowledge, however, that this study does not incorporate animal-health datasets, and its extension to cross-species diagnosis remains an essential direction for future validation and genuine One Health integration.

Recent systematic reviews further emphasize both the promise and current limitations of artificial intelligence (AI) for cutaneous leishmaniasis diagnosis. Prior work has highlighted that only a limited number of studies have addressed microscopy-based diagnosis and that most existing approaches rely on small, single-center datasets [29]. In line with these identified gaps, the present study focuses on probability calibration and lightweight deployment; however, external validation across institutions, microscopes, and animal reservoirs remains an important direction for future work.

Lightweight deep learning models are particularly important for diagnostic applications in endemic and resource-limited settings, where access to high-performance computing infrastructure, stable power supply, and cloud connectivity may be limited. By leveraging a MobileNetV2-based encoder, the proposed framework enables efficient inference with reduced computational and memory requirements, facilitating deployment on standard laboratory computers and mobile or edge devices. This design supports scalable microscopy screening while minimizing operational costs.

The remainder of this paper is organized as follows. The Materials and Methods section describes the dataset, HSV-based pseudo-label generation, data augmentation, class balancing, and model architecture. The Results section presents the experimental findings, including segmentation performance, slide-level discrimination and calibration, operating-point confidence intervals, and statistical analyses. Finally, the Discussion and conclusions section summarizes the main findings, study limitations, and implications for diagnostic reliability and One Health research.

## Materials and methods

### Ethical considerations

This study analyzed publicly available, de-identified microscopy images obtained from a previously published dataset [8]. In the original data collection, ethics approval was granted by the Ethics Committee of Mazandaran University of Medical Sciences (approval number: IR.SUMS.MED.REC.1402.2336), and written informed consent was obtained from all participants.

The present study involved secondary analysis of fully anonymized data released for public research use and did not involve recruitment of new human participants, animal experiments, or access to identifiable personal information. Therefore, additional institutional ethics approval and informed consent were not required.

### Study design and data source

This study employed a publicly available dataset of Giemsa-stained microscopy images from patients clinically suspected of cutaneous leishmaniasis (CL), curated and published in a prior deep-learning investigation [8]. The dataset comprised 292 field-of-view images (138 positive and 154 negative) with slide-level diagnostic annotations. In this dataset, each microscopy image corresponds to a single slide obtained from one patient; no fully digitized whole-slide images or multiple independent fields of view per slide are available. All samples were acquired using an Olympus CX23 light microscope at a native resolution of 224×224  pixels.

The selected U-Net-MobileNetV2 architecture is adopted as a representative lightweight segmentation backbone for this study and is not intended to constitute an exhaustive or optimal comparison against all possible deep learning architectures.

**Fig 1:**
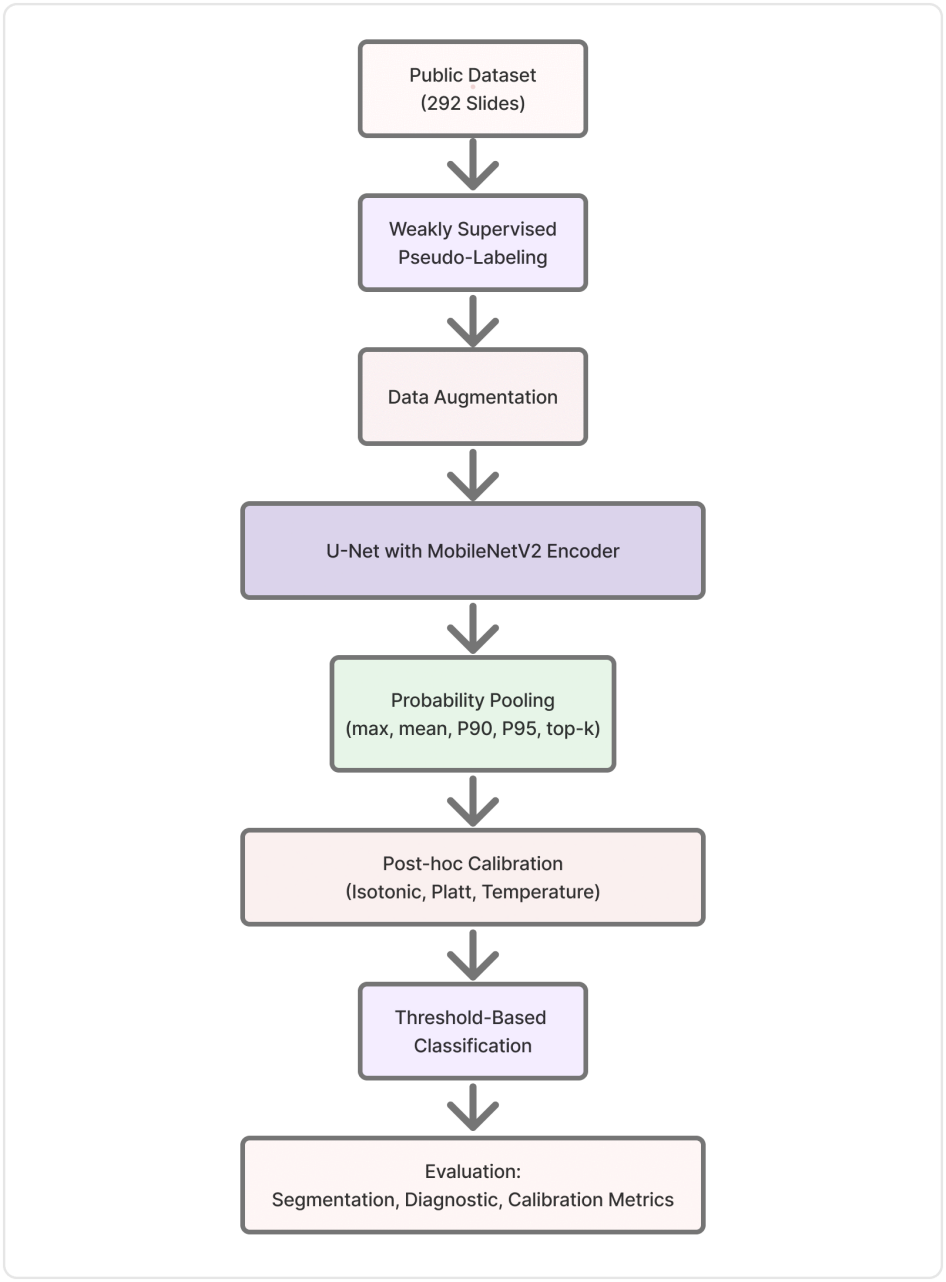
Overview of the proposed lightweight and calibration-aware diagnostic pipeline. Microscopy images from a public dataset were annotated using weakly supervised pseudo-labeling and augmented for training. A U-Net with a MobileNetV2 encoder was trained for pixel-level segmentation. Slide-level diagnostic probabilities were obtained using multiple probability pooling strategies (max, mean, P90, P95, and top-k). Post-hoc calibration was applied using isotonic regression, Platt scaling, and temperature scaling prior to threshold-based classification. Model performance was evaluated using segmentation, diagnostic, and calibration metrics.

Fig 1 provides an overview of the complete analytical pipeline. Public microscopy images were automatically annotated through a weakly supervised pseudo-labeling strategy to approximate parasite regions in positive field-of-view images, compensating for the absence of expert pixel-level annotations. The dataset was expanded via geometric and photometric augmentation and used to train a lightweight U-Net model with a MobileNetV2 encoder optimized for computational efficiency. Following segmentation, slide-level diagnostic probabilities were obtained through probability pooling and refined using post-hoc calibration techniques before final threshold-based classification. A comprehensive set of evaluation metrics was computed to assess segmentation accuracy, diagnostic discrimination, and calibration quality.

### Image preprocessing and pseudo-label generation

Each microscopy image was assigned a slide-level label as positive (containing amastigotes) or negative (without visible amastigotes) according to the dataset-provided diagnostic labels.

For positive samples, binary masks were automatically generated using adaptive thresholding and morphological refinement in the HSV color space to highlight intracellular structures consistent with amastigote morphology. Negative samples were assigned empty masks. Morphological opening and closing with a 3×3  kernel were used to suppress noise and small artifacts.

The use of the HSV color space in this study was motivated by prior evidence showing that color-space transformations can enhance feature discrimination in convolutional neural networks. [30] demonstrated that CNN performance is sensitive to the choice of color space and that non-RGB representations can capture brightness and saturation information that is not well expressed in RGB. Similarly, [31] proposed the UIEC^2^-Net for underwater image enhancement, combining RGB and HSV spaces to improve contrast, luminance, and color stability in complex imaging conditions. Following these findings, HSV was selected here not as a biological proxy but as a color-space heuristic that accentuates chromatic and saturation cues typical of Giemsa staining. The HSV-derived pseudo-labels therefore serve as weak supervision signals to guide the network toward stain-consistent features in the absence of expert pixel-level annotations. Although HSV thresholding appears visually plausible for highlighting parasite-colored regions and is therefore useful for generating weak pixel-level supervision (Fig. 4), it is not suitable as a diagnostic classifier. When used alone as an image-level decision rule, HSV thresholding detects a wide range of non-parasitic structures, including host cell components and staining artifacts, resulting in extremely poor specificity and low overall diagnostic accuracy.

Accordingly, HSV is not used in this study as a diagnostic method, but exclusively as a weak supervision mechanism to guide representation learning. The proposed U-Net–MobileNetV2 model is trained on raw microscopy images and learns discriminative morphological and contextual features that are not explicitly constrained to fixed color rules. This learning-based formulation improves robustness to staining variability and common imaging artifacts, and enables slide-level probability aggregation and post-hoc calibration, which are not achievable using heuristic thresholding alone and are essential for producing clinically interpretable and statistically reliable diagnostic confidence estimates.

### Data augmentation and preparation

To improve generalization and mitigate class imbalance, a targeted augmentation strategy was applied. Each image–mask pair underwent random horizontal and vertical flips, rotations ($90^{\circ }\;$, $180^{\circ }\;$, $270^{\circ }\;$), and controlled contrast and brightness adjustments within clinically realistic limits. All images were resized to 224×224  pixels to match the model input.

This process expanded the dataset to 1,518 augmented positive and 1,540 augmented negative image–mask pairs, producing a near-balanced distribution. The dataset was split at the patient level into 60% training, 20% validation, and 20% testing subsets to ensure independence and prevent data leakage during model development.

For model development, equal-sized subsets of 1,518 augmented image–mask pairs per class were randomly selected to form a balanced training pool. Validation and test sets preserved the original class distribution to ensure unbiased performance evaluation.

Table 1 summarizes the dataset composition and data splits used in this study.

**Table 1:** Summary of dataset composition and data splits used in this study. All diagnostic performance metrics were computed on raw, non-augmented slides. Data augmentation was applied exclusively during model training and did not introduce additional patients or slides.

Item	Count
Total patients	292
Total slides	292
Total microscopy images (raw)	292
Positive slides (with amastigotes)	138
Negative slides (without amastigotes)	154
Augmented positive image–mask pairs (training only)	1,518
Augmented negative image–mask pairs (training only)	1,540
Training set (60%)	Patient-level split
Validation set (20%)	Patient-level split
Test set (20%)	Patient-level split

### Model architecture and training

Model training was performed using the Adam optimizer with parameters $\beta_{1}=0.9\;$ and $\beta_{2}=0.999\;$, a learning rate of $1\times 10^{-4}\;$, and a batch size of 16. The proposed MobileNetV2–U-Net architecture contains 2.69 million parameters, of which 2.31 million are trainable and 0.38 million are non-trainable.

A U-Net architecture was implemented for pixel-wise segmentation, integrating a MobileNetV2 encoder pre-trained on ImageNet to leverage transfer learning for low-level feature extraction. The first 100 layers of the encoder were frozen during the early epochs to preserve generic image representations, while the decoder was fine-tuned for task-specific segmentation.

The decoder comprised sequential upsampling blocks with skip connections, convolutional layers, batch normalization, and ReLU activations [32], progressively reconstructing the segmentation mask at the original spatial resolution. The final layer applied a sigmoid activation [33] to produce per-pixel probabilities between 0 and 1:


$$\sigma (z)=\frac{1}{1+e^{-z}}\;$$ (1)

The network was optimized using the binary cross-entropy loss [34], defined as:


$${ℒ}_{BCE}=-\frac{1}{N}\sum_{i=1}^{N}\left[y^{\left(i\right)}\log({\hat{y}}^{\left(i\right)})+(1-y^{\left(i\right)})\log(1-{\hat{y}}^{\left(i\right)})\right]\;$$ (2)

where ${\hat{y}}^{\left(i\right)}\;$ and $y^{\left(i\right)}\;$ represent the predicted and pseudo-ground-truth labels for pixel *i*, respectively, with $y^{\left(i\right)}\;$ derived from the HSV-based weak-supervision masks described in section “Image Preprocessing and pseudo-label generation.’’ Training was performed using the Adam optimizer with a learning rate of 1 × 10^–4^ for 50 epochs. Early stopping was applied based on the validation Dice coefficient, with Intersection-over-Union (IoU) monitored as a secondary performance metric.

The held-out test subset was reserved exclusively for the final, unbiased evaluation of segmentation and slide-level diagnostic performance, with slide probabilities later derived through pooling and post-hoc calibration as described in section “Slide-level probability pooling and calibration.”

### Slide-level probability pooling and calibration

Pixel-wise probability maps produced by the segmentation model were converted into a single slide-level diagnostic confidence score using a pooling-based strategy. The *maximum* predicted probability across all pixels was selected as the pooling function, given its sensitivity to small or focal infections and its ability to minimize false negatives during screening. This approach ensures that even sparse amastigote clusters contribute meaningfully to the final diagnostic decision.

To improve interpretability and reliability of these probabilistic outputs, post-hoc calibration was applied using three established techniques: *isotonic regression* [25], *Platt scaling*, and *temperature scaling*. Each calibration model was fitted on the validation set and subsequently applied to the independent test set to prevent data leakage and ensure statistical independence.

Calibration performance was quantified using the Brier score, Expected Calibration Error (ECE), and Maximum Calibration Error (MCE), supported by reliability diagrams for visual assessment. Statistical significance of calibration improvement was evaluated using the Wilcoxon signed-rank test on paired Brier scores. Differences in discriminative ability before and after calibration were assessed using the DeLong test for correlated receiver operating characteristic (ROC) curves.

### Evaluation metrics

Model performance was assessed at two complementary levels: pixel-wise segmentation accuracy and slide-level diagnostic reliability.

At the *pixel level*, metrics quantified the morphological agreement of predicted segmentation masks against pseudo-label references [35,36]. At the *slide level*, pooled probabilities ($p_{i}\;$) were evaluated for diagnostic discrimination and calibration using threshold-based classification.

Pixel-level metrics are reported for descriptive assessment of segmentation behavior; however, statistical inference and confidence intervals are computed exclusively at the slide level to avoid violating independence assumptions.

Discrimination was measured via the Area Under the Receiver Operating Characteristic curve (AUROC) and the Area Under the Precision–Recall curve (AUPRC), while calibration quality was assessed through the Brier score, Expected Calibration Error (ECE), and Maximum Calibration Error (MCE), complemented by reliability diagrams.

All metrics followed standard biomedical imaging conventions. Confidence intervals for proportion-based metrics (e.g., sensitivity, specificity, precision, and NPV) were estimated using Wilson intervals, whereas those for AUROC, AUPRC, Brier score, and ECE were obtained from 1,000 bootstrap resamples.

Evaluation metrics used at the pixel and slide levels are summarized in Table 2.

**Table 2:** Summary of evaluation metrics used at the pixel and slide levels.

Metric	Definition	Level
Accuracy	$\frac{TP+TN}{TP+TN+FP+FN}\;$	Pixel, Slide
Precision (PPV)	$\frac{TP}{TP+FP}\;$	Pixel, Slide
Recall (Sensitivity)	$\frac{TP}{TP+FN}\;$	Pixel, Slide
Specificity (TNR)	$\frac{TN}{TN+FP}\;$	Pixel, Slide
Negative Predictive Value (NPV)	$\frac{TN}{TN+FN}\;$	Slide
Intersection over Union (IoU)	$\frac{TP}{TP+FP+FN}\;$	Pixel
Dice Similarity Coefficient	$\frac{2TP}{2TP+FP+FN}\;$	Pixel
AUROC	Area under the ROC curve (bootstrapped 95% CI).	Slide
AUPRC	Area under the precision–recall curve (bootstrapped 95% CI).	Slide
Brier Score	$\frac{1}{n}\sum_{i=1}^{n}(p_{i}-y_{i}{)}^{2}\;$	Slide
Expected Calibration Error (ECE)	Weighted difference between predicted and observed frequencies.	Slide
Maximum Calibration Error (MCE)	Maximum absolute difference between predicted and observed frequencies.	Slide

Sensitivity and specificity were estimated at the slide level on the independent test set, where true positives (TP) denote correctly identified microscopy-positive slides and true negatives (TN) denote correctly identified microscopy-negative slides.

### Statistical analysis

To evaluate whether post-hoc calibration led to systematic improvements in probability reliability at the slide level, paired statistical comparison was conducted using the Wilcoxon signed-rank test. This non-parametric test compares paired observations by assessing whether the median of the differences between paired samples deviates from zero.

Given paired Brier scores $\{b_{i}^{\text{uncal}},b_{i}^{\text{cal}}{\}}_{i=1}^{n}\;$ for *n* slides, the signed differences are defined as


$$d_{i}=b_{i}^{\text{cal}}-b_{i}^{\text{uncal}}.\;$$ (3)

After removing zero differences, the absolute values $|d_{i}|\;$ are ranked, and the Wilcoxon test statistic *W* is computed as the sum of ranks corresponding to positive signed differences. This statistic quantifies whether calibration is associated with a systematic change in probability error across slides.

The Wilcoxon signed-rank test was used as a distribution-free approach appropriate for paired slide-level measurements.

### Results and discussion

This section reports the performance of the proposed U-Net–MobileNetV2 framework for automated parasite segmentation and slide-level classification of cutaneous leishmaniasis (CL) microscopy images. Results are presented for the training, validation, and independent test sets, with emphasis on segmentation behavior relative to HSV-derived pseudo-labels, diagnostic consistency, and generalization stability.

### Training and validation performance

**Fig 2:**
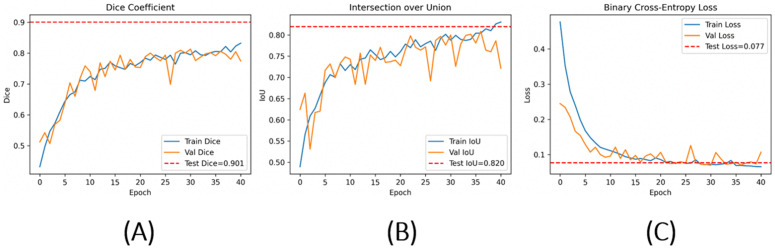
Training and validation performance of the U-Net–MobileNetV2 model. **(A)** Dice coefficient, **(B)** Intersection-over-Union (IoU), and **(C)** binary cross-entropy loss over 50 epochs. Solid blue and orange curves represent training and validation performance, respectively. Dashed red lines denote final independent test-set performance (Dice = 0.901, IoU = 0.820, loss = 0.077). The close alignment between training and validation curves indicates stable optimization under weak supervision.

Fig 2 illustrates the evolution of Dice coefficient, Intersection-over-Union (IoU), and binary cross-entropy loss over 50 training epochs. Overall, the model demonstrates smooth and stable convergence, with no evidence of abrupt divergence or training instability.

Binary cross-entropy loss decreased rapidly during the early epochs and stabilized at approximately 0.07 for both training and validation sets, indicating effective optimization. The close correspondence between training and validation curves across Dice, IoU, and loss suggests limited overfitting despite the small dataset size and confirms the effectiveness of the applied regularization and augmentation strategy.

Because expert-annotated pixel-level ground truth was unavailable, segmentation metrics quantify agreement with HSV-based pseudo-labels rather than absolute biological parasite boundaries. Accordingly, these results are interpreted as indicators of internal learning consistency and stain-aware feature extraction rather than definitive measures of true parasite morphology.

At epoch 40, which corresponded to the best validation performance prior to degradation, the model achieved a training Dice coefficient of 0.829 and IoU of 0.835. Validation Dice and IoU reached 0.805 and 0.786, respectively, with corresponding binary cross-entropy losses of 0.069 (training) and 0.073 (validation). Training and validation accuracies were 0.971 and 0.969, respectively. Precision and recall remained balanced across splits (precision ≈ 0.92; recall ≈ 0.92), while specificity consistently exceeded 0.97.

### Independent test performance

On the held-out independent test set, the model achieved a Dice coefficient of 0.901 and an IoU of 0.820, with a binary cross-entropy loss of 0.077. Slide-level accuracy reached 0.968, accompanied by a sensitivity of 0.890, specificity of 0.983, and precision of 0.912.

Importantly, the Dice score observed on the independent test set (0.901) is consistent with the final training and validation trends, indicating stable convergence of the optimization process. The absence of divergence between training and validation curves across epochs suggests that the model maintains generalization under weak supervision despite the limited dataset size, as illustrated in Fig 2. These results confirm that the lightweight MobileNetV2-based encoder effectively captures stain-consistent parasitic patterns across unseen microscopy images without evidence of pronounced overfitting.

### Independent test performance

The proposed U-Net–MobileNetV2 model demonstrated stable generalization on the independent test set, as summarized in Table 3. At the pixel level, agreement with HSV-based pseudo-label references yielded a Dice coefficient of 0.901 and an Intersection-over-Union (IoU) of 0.820, indicating consistent segmentation behavior under the adopted weak-supervision scheme.

**Table 3:** Segmentation and classification performance across training, validation, and independent test sets.

Metric	Training	Validation	Test (held-out)
Accuracy	0.972	0.970	0.968
IoU (binary)	0.822	0.808	0.820
Dice coefficient	0.822	0.819	0.901
Precision	0.916	0.944	0.912
Recall (Sensitivity)	0.921	0.869	0.890
Specificity	0.981	0.989	0.983
Loss (BCE)	0.0648	0.0766	0.0767

Test accuracy reached 0.968, with a precision of 0.912 and a recall (sensitivity) of 0.890, reflecting a balanced trade-off between false-positive suppression and detection sensitivity. The model further achieved a specificity of 0.983, highlighting effective discrimination of parasite-negative regions and control of spurious detections.

It is important to emphasize that these segmentation metrics quantify consistency with heuristic pseudo-labels rather than biologically validated parasite boundaries. Accordingly, the reported Dice and IoU values should be interpreted as indicators of internal model consistency and stain-aware feature learning rather than absolute biological segmentation accuracy. Nevertheless, the close agreement between test performance and training–validation trends suggests stable learning dynamics without evidence of pronounced overfitting, despite the limited dataset size.

Overall, the lightweight U-Net–MobileNetV2 configuration exhibits reliable and reproducible performance under weak supervision, providing a robust foundation for subsequent slide-level probability pooling and calibration analyses.

**Fig 3:**
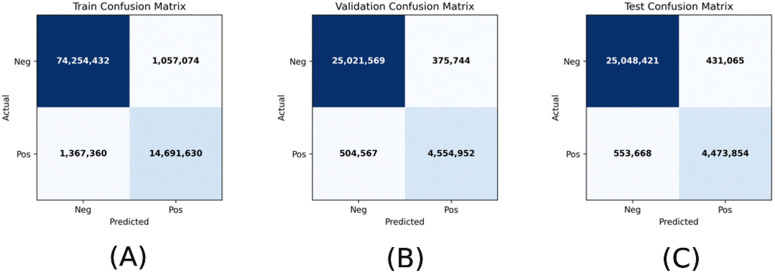
Pixel-level confusion matrices for model evaluation. **(A)** Training set confusion matrix. **(B)** Validation set confusion matrix. **(C)** Independent test set confusion matrix. True positives (TP), false positives (FP), false negatives (FN), and true negatives (TN) are shown. Confusion patterns are consistent across data splits, indicating stable learning dynamics under weak supervision.

Fig 3 presents the pixel-level confusion matrices for the training, validation, and independent test sets. Across all data partitions, the model correctly classifies a large proportion of parasite-positive and parasite-negative pixels, indicating stable behavior under the adopted weak-supervision framework.

On the independent test set, the model produced 4,473,854 true-positive pixels and 25,048,421 true-negative pixels, alongside 431,065 false-positive and 553,668 false-negative predictions. This error distribution corresponds to high specificity (0.983) with a non-negligible number of false-negative pixels, consistent with the reported sensitivity (0.890). The relative balance between false-positive and false-negative errors suggests that the model does not exhibit a strong bias toward either class.

It should be noted that these confusion matrices are derived from pixel-wise comparisons against HSV-based pseudo-labels rather than expert-annotated ground truth. As such, they primarily characterize internal consistency, class-separation behavior, and learning stability rather than definitive biological correctness. Nevertheless, the similarity of confusion patterns across training, validation, and test sets indicates reproducible performance and no evidence of pronounced overfitting despite the limited dataset size.

Following pixel-level segmentation analysis, slide-level probability pooling and post-hoc calibration were applied to aggregate pixel-wise predictions into clinically interpretable diagnostic confidence scores. Calibration performance and reliability analyses are presented in the subsequent sections.

**Fig 4:**
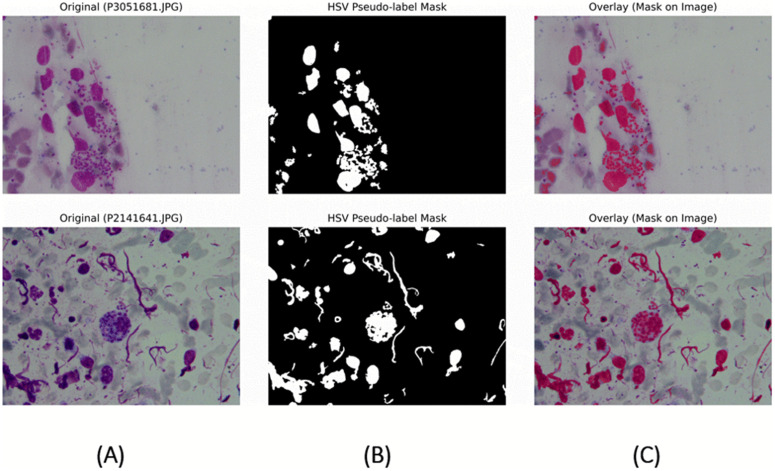
Representative examples of HSV-based pseudo-label quality used for weak supervision. **(A)** Original Giemsa-stained microscopy image. **(B)** HSV-derived pseudo-label mask. **(C)** Overlay of the mask on the original image. Examples include typical parasite-containing regions as well as challenging cases where stain artifacts, host cell structures, or debris are partially captured. These visualizations highlight both the utility and limitations of heuristic pseudo-labeling and motivate cautious interpretation of pixel-level segmentation metrics.

Fig 4 illustrates representative examples of the HSV-based labeling strategy used for weak supervision. For each case, the figure shows the original microscopy-positive Giemsa-stained image, the corresponding HSV-derived pseudo-label mask, and the overlay of the mask on the original image. Examples include typical cases in which the derived masks align well with intracellular parasite clusters, as well as challenging cases where non-parasitic structures such as stain debris, host cell components, or elongated artifacts are partially highlighted. These examples illustrate the heuristic nature of the generated labels, which may include both false-positive and incomplete regions. Consequently, segmentation metrics reported in this study reflect agreement with stain-consistent proxy labels rather than expert-verified parasite boundaries. The inclusion of these qualitative examples is intended to provide transparency regarding the strengths and limitations of the weak supervision scheme.

### Comparative performance of calibration methods

Following pixel-level segmentation, slide-level diagnostic probabilities were obtained via probability pooling, using the maximum predicted pixel probability per slide. This aggregation strategy prioritizes sensitivity to sparse parasite clusters, ensuring that localized infections contribute to the final diagnostic score. The resulting slide-level probabilities were subsequently calibrated using three post-hoc methods: isotonic regression, Platt scaling, and temperature scaling.

Calibration performance was evaluated on the independent test set using the Brier score, Expected Calibration Error (ECE), and standard discrimination metrics. As summarized in Table 4, the uncalibrated model exhibited strong discriminative ability (AUROC = 0.9829; AUPRC = 0.999) but demonstrated substantial miscalibration, with a Brier score of 0.089 and ECE of 0.120, indicating overconfident probability estimates.

**Table 4:** Comparative performance of post-hoc calibration methods on the independent test set. Calibration metrics are reported at the slide level.

Method	Brier ↓	ECE ↓	AUROC ↑	Sensitivity	Specificity	Accuracy	PPV
Uncalibrated	0.0886	0.1204	0.9829	0.8810	1.0000	0.8880	1.0000
Isotonic	0.0298	0.0230	0.9782	0.9860	0.5560	0.9610	0.9720
Platt	0.0390	0.0460	0.9829	1.0000	0.0000	0.9410	0.9410
Temperature	0.0840	0.1555	0.9829	0.8810	1.0000	0.8880	1.0000

Isotonic regression produced the largest improvement in calibration quality, reducing the Brier score to 0.030 and ECE to 0.023, corresponding to relative reductions of approximately 66% and 81%, respectively. Platt scaling achieved moderate calibration improvement (Brier = 0.039; ECE = 0.046), whereas temperature scaling did not improve calibration and resulted in increased ECE (0.156).

Notably, post-hoc adjustment did not significantly affect discriminative performance. DeLong’s test comparing AUROC values before and after isotonic calibration showed no statistically significant difference (AUROC$\backslash {(}_{\text{uncal}}\;$ = 0.9829 vs. AUROC$\backslash {(}_{\text{iso}}\;$ = 0.9782; *Δ* = 0.0047; p=0.5065 ), indicating that probability calibration improved reliability without compromising ranking performance.

Bootstrap resampling (1,000 iterations) confirmed the robustness of these findings. For isotonic calibration, the AUROC was 0.9787 [0.9517–0.9975], the Brier score was 0.0294 [0.0089–0.0548], and the ECE was 0.0258 [0.0064–0.0528]. In contrast, the uncalibrated model exhibited substantially higher and less stable calibration errors (ECE = 0.1215 [0.0807–0.1696]).

At the default decision threshold of 0.5, isotonic calibration yielded a sensitivity of 0.9860 and specificity of 0.5560, reflecting a deliberate shift toward minimizing missed infections at the expense of increased false positives. This operating profile is appropriate for screening-oriented use cases, whereas the uncalibrated and temperature-scaled models, which maintained perfect specificity, are better suited for confirmatory settings. Platt scaling achieved perfect sensitivity but failed to identify negative cases, limiting its practical utility.

Overall, isotonic regression provided the most reliable balance between probabilistic calibration and diagnostic discrimination within the proposed framework.

From a clinical perspective, at the diagnostic threshold of 0.5, the isotonic-calibrated model achieved a screening-oriented operating profile characterized by very high sensitivity (0.9860), minimizing missed infections, alongside moderate specificity (0.5560). This trade-off resulted in the highest overall accuracy among the evaluated calibration strategies (0.9610). Such a profile is particularly appropriate for diagnostic screening settings, where reducing false negatives is prioritized to enable timely treatment and disease control.

Although Platt scaling achieved perfect sensitivity, it shifted calibrated probabilities above the default decision threshold, resulting in zero true-negative predictions (specificity = 0.0) and limiting its practical utility. In contrast, the uncalibrated and temperature-scaled models preserved perfect specificity but exhibited lower sensitivity, increasing the risk of missed infections. Taken together, these findings indicate that isotonic regression provides the most balanced and clinically interpretable calibration strategy for screening-oriented microscopy-based cutaneous leishmaniasis diagnostics under the proposed framework.

### Ablation study of slide-level pooling strategies

To assess the robustness of slide-level probability aggregation, we conducted an ablation study comparing multiple pooling strategies, including max pooling, mean pooling, percentile-based pooling (P90 and P95), log-sum-exp pooling, and top-*k* mean pooling. This analysis was performed on the independent test set using identical segmentation outputs to isolate the effect of the aggregation method.

As summarized in Table 5, max pooling achieved the highest sensitivity (1.000) and AUROC (1.000), indicating superior ability to detect sparse parasite clusters.Top-5 mean pooling produced comparable discrimination performance with a slightly lower Brier score; however, max pooling was retained due to its simplicity and consistently high sensitivity. In contrast, mean pooling and lower-percentile strategies substantially reduced sensitivity, while log-sum-exp pooling exhibited unstable behavior and failed to discriminate between positive and negative slides.

**Table 5:** Ablation study of slide-level probability pooling strategies on the independent test set. Metrics are reported at the slide level.

Pooling Strategy	AUROC	Brier	Sensitivity	Remarks
Max pooling	1.000	0.031	1.000	Selected (high sensitivity)
Top-5 mean pooling	1.000	0.030	1.000	Comparable performance
P95 pooling	0.983	0.089	0.881	Reduced sensitivity
P90 pooling	0.967	0.225	0.713	Reduced sensitivity
Mean pooling	0.990	0.657	0.007	Poor detection of positives
Log-sum-exp pooling	0.500	0.059	1.000	Unstable / non-discriminative

These findings support max pooling as an appropriate aggregation strategy for screening-oriented microscopy-based diagnosis, where sensitivity to sparse parasite signals is clinically prioritized. As shown in Table 5, max pooling and top-5 mean pooling achieved comparable discrimination and calibration, whereas percentile and mean pooling reduced sensitivity substantially on this dataset. Therefore, max pooling was selected as a simple and effective operating choice under the study’s screening objective.

### Visual analysis of calibration and diagnostic performance

**Fig 5:**
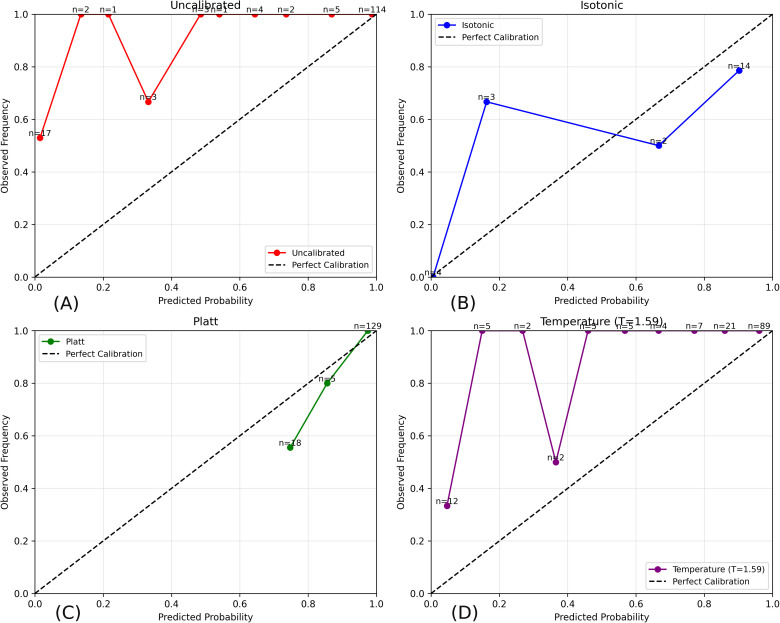
Reliability diagrams for slide-level probability calibration. **(A)** Uncalibrated model. **(B)** Isotonic regression. **(C)** Platt scaling. **(D)** Temperature scaling. The dashed diagonal represents perfect calibration. Isotonic regression achieves the closest alignment with this reference line, indicating improved correspondence between predicted probabilities and observed infection frequencies.

The quantitative improvements achieved through post-hoc calibration are further illustrated through reliability diagrams in Fig 5. Reliability diagrams compare predicted probabilities with observed outcome frequencies; deviation from the diagonal indicates miscalibration, where predictions are either overconfident (points above the diagonal) or underconfident (points below the diagonal).

The uncalibrated model exhibits pronounced overconfidence across most probability bins, particularly at lower predicted probabilities, where observed infection frequencies substantially exceed predicted values. This behavior explains the elevated Expected Calibration Error (ECE = 0.120) and indicates that raw network outputs are poorly aligned with true outcome frequencies despite strong discriminative performance.

Isotonic regression substantially improves calibration by reshaping the probability distribution to better reflect empirical outcome frequencies. The isotonic curve lies closer to the diagonal across bins, demonstrating improved agreement between predicted confidence and observed infection rates and visually corroborating the marked reduction in both Brier score and ECE.

Platt scaling partially mitigates overconfidence at higher probability bins but compresses predictions toward extreme values, leading to limited discrimination of negative cases and unstable calibration behavior. Temperature scaling, while preserving ranking performance, introduces irregular bin-wise deviations and fails to correct systematic overconfidence, consistent with its increased ECE.

Beyond improved calibration, this behavior also promotes probability polarization, whereby predictions concentrate toward 0 or 1, enhancing clinical interpretability by distinguishing confidently negative from confidently positive slides. Overall, these visualizations confirm that isotonic regression yields the most reliable and interpretable probability estimates among the evaluated methods, supporting its use in screening-oriented diagnostic workflows where calibrated confidence is critical for decision-making.

While the ROC curves remain highly similar, the probability density distributions reveal a clear redistribution of predicted probabilities after isotonic calibration. Calibrated outputs exhibit increased polarization toward the extremes of 0 and 1, reflecting improved separation between confidently negative and confidently positive cases. This behavior enhances probability interpretability and reliability rather than discriminative power.

Together, these results demonstrate that isotonic calibration improves the clinical interpretability of predicted probabilities while maintaining near-identical discriminative performance

**Fig 6:**
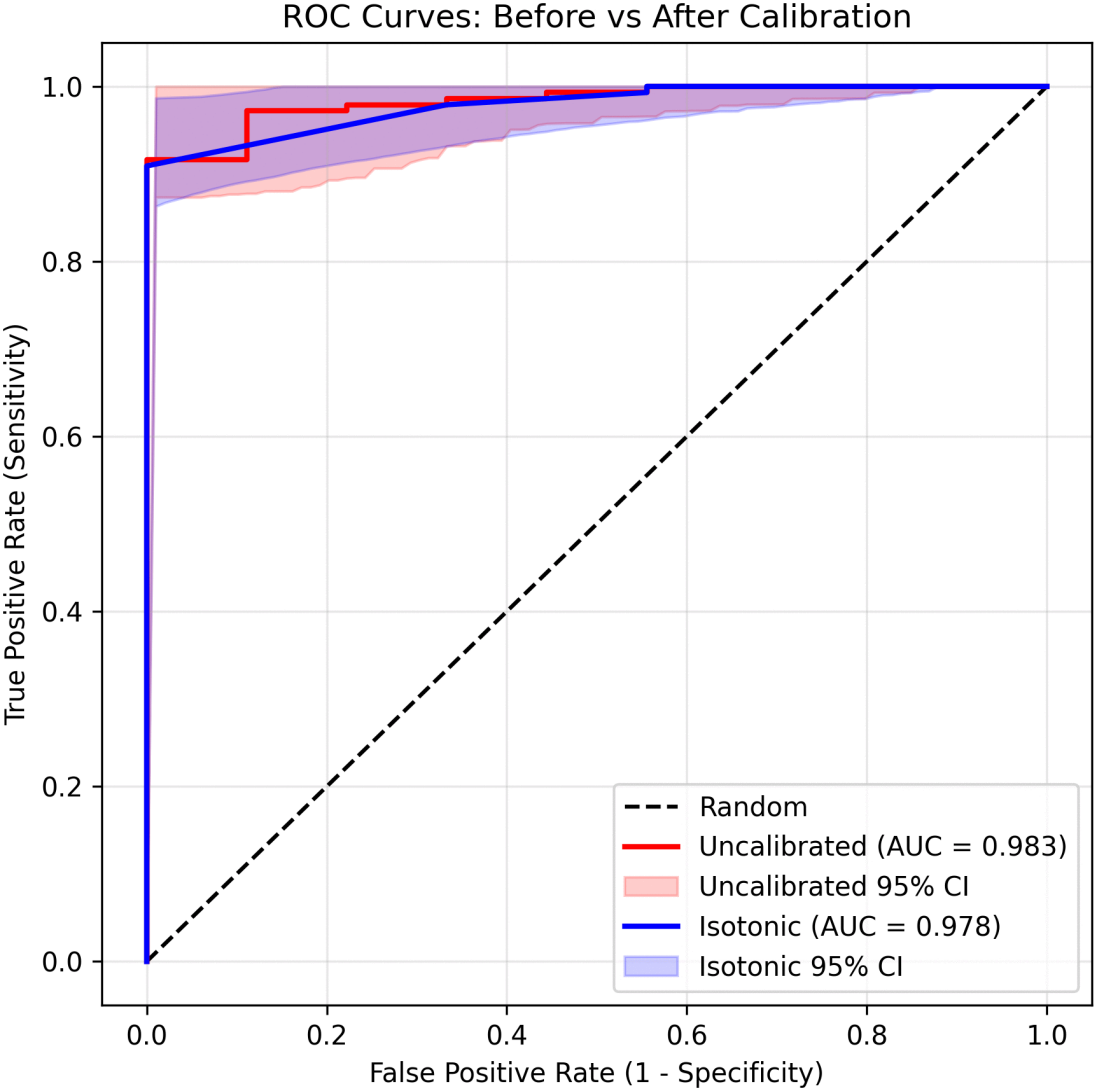
Receiver operating characteristic (ROC) curves with 95% confidence intervals for the uncalibrated (red) and isotonic-calibrated (blue) models. Discrimination performance is preserved after calibration (AUROC$\backslash {(}_{\text{uncal}}\;$ = 0.983; AUROC$\backslash {(}_{\text{iso}}\;$ = 0.978).

Fig 6 compares ROC curves before and after isotonic calibration. As expected for post-hoc calibration, discrimination performance was preserved, with AUROC values of 0.983 for the uncalibrated model and 0.978 after calibration. DeLong’s test confirmed that this difference was not statistically significant (p=0.5065 ), indicating that calibration did not meaningfully affect ranking performance.

**Fig 7:**
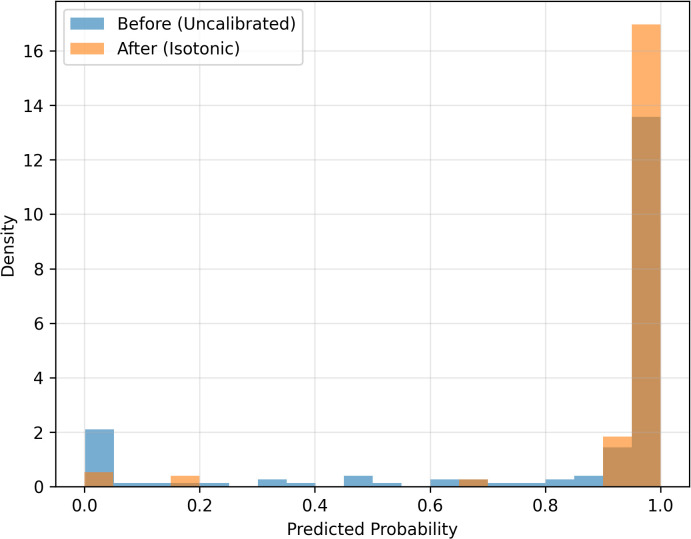
Distribution of predicted slide-level infection probabilities before (blue) and after isotonic calibration (orange). Calibration redistributes predictions toward extreme values, improving confidence interpretability while preserving overall discrimination.

Fig 7 illustrates the distribution of predicted probabilities before and after isotonic calibration. While uncalibrated predictions are dispersed across intermediate probability values, isotonic calibration produces a clearer concentration toward extreme probabilities near 0 and 1. This probability polarization reflects improved separation between confidently negative and confidently positive cases, enhancing interpretability without increasing discriminative power.

**Fig 8:**
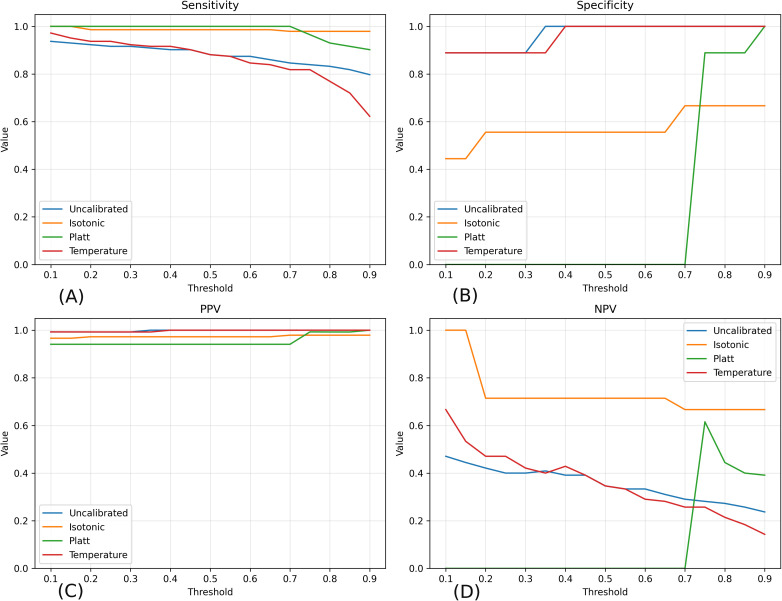
Variation of key diagnostic metrics across decision thresholds. **(A)** Sensitivity. **(B)** Specificity. **(C)** Positive predictive value (PPV). **(D)** Negative predictive value (NPV). Curves are shown for uncalibrated, isotonic, Platt, and temperature-scaled models. The figure illustrates operating-point trade-offs relevant to clinical screening, with isotonic regression demonstrating the most stable diagnostic behavior across thresholds.

To assess diagnostic robustness across varying decision criteria, Fig 8 illustrates trends in sensitivity, specificity, positive predictive value (PPV), and negative predictive value (NPV) as a function of the decision threshold. Isotonic regression consistently maintained high sensitivity (generally exceeding 0.95) across a broad range of thresholds, indicating strong robustness for screening-oriented use cases.

PPV remained high across all models and thresholds, reflecting a low false-positive rate in this dataset; however, the associated confidence intervals (reported separately) indicate increased uncertainty due to the limited number of negative cases. In contrast, NPV decreased progressively at higher thresholds, consistent with the expected trade-off between sensitivity and specificity as decision criteria become more stringent.

Overall, isotonic calibration exhibited the most stable and interpretable balance between sensitivity and specificity across thresholds, supporting its suitability for clinical screening scenarios where minimizing missed infections is prioritized while maintaining reasonable false-alarm rates.

To statistically assess whether calibration improved probability reliability at the slide level, a paired Wilcoxon signed-rank test was performed on per-slide Brier scores between the uncalibrated and isotonic-calibrated models. The analysis demonstrated a statistically significant reduction in Brier score following isotonic calibration (Wilcoxon W=790 , p<0.001 ), confirming that the observed improvement in calibration performance is robust and not attributable to random variation. The use of a non-parametric test is particularly appropriate given the limited sample size and non-Gaussian distribution of probability errors.

Table 6 reports slide-level sensitivity, specificity, PPV, and NPV together with Wilson 95% confidence intervals. While point estimates of PPV are high across all models, the associated confidence intervals provide a more informative assessment of diagnostic reliability by quantifying uncertainty arising from the limited number of negative slides. In particular, specificity and NPV exhibit wider confidence intervals, reflecting the small negative-class sample size and the sensitivity of these metrics to a few misclassified cases. In contrast, sensitivity estimates show comparatively narrower intervals, indicating stable detection of positive slides. Reporting confidence intervals alongside point estimates therefore enables a more transparent and statistically robust interpretation of diagnostic performance at the slide level.

**Table 6:** Slide-level diagnostic performance with Wilson 95% confidence intervals on the independent test set.

Method	Sensitivity (95% CI)	Specificity (95% CI)	PPV (95% CI)	NPV (95% CI)
Uncalibrated	0.881 [0.818–0.924]	1.000 [0.701–1.000]	1.000 [0.970–1.000]	0.346 [0.194–0.538]
Isotonic	0.986 [0.950–0.996]	0.556 [0.267–0.811]	0.972 [0.931–0.989]	0.714 [0.359–0.918]
Platt	1.000 [0.974–1.000]	0.000 [0.000–0.299]	0.941 [0.891–0.969]	0.000 [0.000–0.000]
Temperature	0.881 [0.818–0.924]	1.000 [0.701–1.000]	1.000 [0.970–1.000]	0.346 [0.194–0.538]

### Summary of key findings

In summary, the proposed U-Net–MobileNetV2 architecture demonstrated stable and consistent segmentation performance for *Leishmania* parasite detection under a weakly supervised setting, achieving a Dice coefficient of 0.901 and an Intersection-over-Union of 0.820 on the independent test set when evaluated against HSV-derived pseudo-labels. These results indicate robust learning dynamics and good generalization across held-out microscopy slides, while acknowledging that segmentation accuracy reflects agreement with heuristic labels rather than expert-annotated ground truth.

At the slide level, post-hoc probability calibration substantially improved the reliability of diagnostic predictions. Among the evaluated methods, isotonic regression achieved the best calibration performance, reducing the Brier score from 0.089 (uncalibrated) to 0.030 and the Expected Calibration Error (ECE) from 0.120 to 0.023, while preserving strong discriminative performance (AUROC = 0.9782). Statistical analysis using paired Wilcoxon signed-rank testing confirmed that the improvement in probability reliability was significant (p<0.001 ), and DeLong’s test indicated no meaningful degradation in ranking performance (p=0.5065 ).

From a clinical perspective, the isotonic-calibrated model exhibited an optimal screening-oriented operating profile, achieving high sensitivity (0.9860) with moderate specificity (0.5560) and an overall accuracy of 0.961 at the default decision threshold. This balance prioritizes minimization of missed infections, which is critical for early detection and disease control in endemic settings.

Collectively, these findings support a complete and interpretable diagnostic pipeline that integrates lightweight deep learning–based segmentation, biologically motivated slide-level aggregation, and statistically validated probability calibration. While the current results are limited to a single-center human microscopy dataset, the proposed framework provides a reliable foundation for scalable microscopy-based cutaneous leishmaniasis screening and future extension toward broader One Health validation.

## Limitations

First, this study relies on a relatively small dataset comprising 292 field-of-view microscopy images acquired from a single center using a single microscope model. This reflects the current lack of large, publicly available, curated datasets for cutaneous leishmaniasis microscopy, for which no widely adopted open-access benchmarks currently exist. Although patient-level splitting and data augmentation were applied to reduce bias and improve robustness, the homogeneous acquisition conditions may limit generalizability to other laboratories, staining protocols, and epidemiological settings. Accordingly, the present results should be interpreted as a proof-of-concept demonstrating methodological feasibility rather than evidence for immediate clinical deployment.

Second, parasite segmentation was learned using HSV-derived pseudo-labels due to the absence of expert pixel-level annotations. Consequently, segmentation metrics such as Dice and IoU reflect agreement with heuristic masks rather than biologically confirmed parasite boundaries. This weak-supervision strategy enables scalable model development under realistic annotation constraints, but expert-annotated subsets would be valuable for future validation.

Third, although probability calibration substantially improved reliability (reducing the Expected Calibration Error from 0.119 to 0.023), calibration and statistical validation were performed on data drawn from the same cohort. Further validation on larger, multi-center datasets is required to assess robustness across different staining protocols, microscopy systems, and clinical settings, and to support future integration within a broader One Health framework.

## Conclusion

This study presents a lightweight, calibration-aware deep learning framework for automated analysis of Giemsa-stained microscopy images in cutaneous leishmaniasis diagnosis. By integrating a U-Net architecture with a MobileNetV2 encoder, the proposed model achieved stable segmentation performance under weak supervision, with a Dice coefficient of 0.901 and an Intersection-over-Union of 0.820 on an independent test set when evaluated against HSV-derived pseudo-labels. At the slide level, the framework demonstrated strong discriminative capability (AUROC = 0.978), supporting reliable separation between infected and non-infected samples.

Post-hoc probability calibration using isotonic regression substantially improved diagnostic reliability, reducing the Brier score from 0.089 to 0.030 and the Expected Calibration Error from 0.120 to 0.023 without significantly affecting discrimination performance. These calibrated outputs provide more interpretable and trustworthy probability estimates, which are essential for clinical decision support, particularly in screening-oriented diagnostic workflows.

From a broader perspective,this work contributes a resource-efficient and interpretable AI framework that is well suited as a proof-of-concept for microscopy-based screening in endemic and resource-limited settings. While the present study is limited to human microscopy data, the proposed approach aligns conceptually with the One Health paradigm given the zoonotic nature of cutaneous leishmaniasis. Future work will focus on external multi-center validation, incorporation of expert annotations, and extension toward animal reservoirs where appropriate, as well as mobile and cloud-based implementations to support scalable diagnostic screening and disease control efforts.

### Ethics approval and consent to participate

The microscopy images analyzed in this study were obtained from a publicly available dataset originally published by [8]. In the original study, ethics approval was granted by the Ethics Committee of Mazandaran University of Medical Sciences (approval number: IR.SUMS.MED.REC.1402.2336), and written informed consent was obtained from all participants.

The present study involved secondary analysis of fully anonymized human data released for public research use and did not involve recruitment of new human participants or access to identifiable personal information. Therefore, additional institutional ethics approval and informed consent to participate were not required.

## Abbreviations

CL: Cutaneous Leishmaniasis
VL: Visceral Leishmaniasis
MCL: Mucocutaneous Leishmaniasis
AI: Artificial Intelligence
FN: False Negative
FP: False Positive
TN: True Negative
TP: True Positive
U-Net: Encoder–decoder CNN with skip connections for segmentation
BCE: Binary Cross-Entropy
IoU: Intersection over Union
AUC: Area Under the ROC Curve
AUROC: Area Under the Receiver Operating Characteristic curve
AUPRC: Area Under the Precision–Recall Curve
ReLU: Rectified Linear Unit
HSV: Hue–Saturation–Value color space
Giemsa stain: Romanowsky-type stain for blood and parasite smears
PPV: Positive Predictive Value
NPV: Negative Predictive Value
Sigmoid: Logistic activation function
Brier: Brier score (mean squared error of predicted probabilities).
